# Molecular Biomarkers of Neurodegenerative Disorders: A Practical Guide to Their Appropriate Use and Interpretation in Clinical Practice

**DOI:** 10.3390/ijms25084323

**Published:** 2024-04-13

**Authors:** Luisa Agnello, Caterina Maria Gambino, Anna Maria Ciaccio, Anna Masucci, Roberta Vassallo, Martina Tamburello, Concetta Scazzone, Bruna Lo Sasso, Marcello Ciaccio

**Affiliations:** 1 Institute of Clinical Biochemistry, Clinical Molecular Medicine, and Clinical Laboratory Medicine, Department of Biomedicine, Neurosciences and Advanced Diagnostics, University of Palermo, 90127 Palermo, Italy; luisa.agnello@unipa.it (L.A.); caterinamaria.gambino@unipa.it (C.M.G.); anna.masucci@community.unipa.it (A.M.); roberta.vassallo03@community.unipa.it (R.V.); martina.tamburello@community.unipa.it (M.T.); concetta.scazzone@unipa.it (C.S.); bruna.losasso@unipa.it (B.L.S.); 2 Department of Laboratory Medicine, University Hospital “P. Giaccone”, 90127 Palermo, Italy; 3 Internal Medicine and Medical Specialties “G. D’Alessandro”, Department of Health Promotion, Maternal and Infant Care, University of Palermo, 90127 Palermo, Italy; annamaria.ciaccio@unipa.it

**Keywords:** neurodegeneration, Alzheimer’s disease, Parkinson’s disease, biomarker, laboratory medicine

## Abstract

Neurodegenerative disorders (NDs) represent a group of different diseases characterized by the progressive degeneration and death of the nervous system’s cells. The diagnosis is challenging, especially in the early stages, due to no specific clinical signs and symptoms. In this context, laboratory medicine could support clinicians in detecting and differentiating NDs. Indeed, biomarkers could indicate the pathological mechanisms underpinning NDs. The ideal biofluid for detecting the biomarkers of NDs is cerebrospinal fluid (CSF), which has limitations, hampering its widespread use in clinical practice. However, intensive efforts are underway to introduce high-sensitivity analytical methods to detect ND biomarkers in alternative nonivasive biofluid, such as blood or saliva. This study presents an overview of the ND molecular biomarkers currently used in clinical practice. For some diseases, such as Alzheimer’s disease or multiple sclerosis, biomarkers are well established and recommended by guidelines. However, for most NDs, intensive research is ongoing to identify reliable and specific biomarkers, and no consensus has yet been achieved.

## 1. Introduction

Neurodegenerative disorders (NDs) are diseases characterized by a gradual selective neuronal loss in specific brain areas [1]. NDs represent a major and increasing global public health concern, accounting for a significant portion of the disease burden worldwide [2]. The World Health Organization (WHO) estimated that within 15–20 years, NDs will become the second-leading cause of death, after cardiovascular diseases, owing to the constant increase in the elderly population [3]. Indeed, aging is the most important risk factor for NDs. Beyond age, several genetic and environmental factors contribute to the ND pathogenesis. Some NDs are familial, being associated with causative genes, but most cases are sporadic, with yet-unknown etiology.

The clinical features of NDs, especially in the early stages, differ according to the anatomic region involved. Based on clinical features, NDs can be classified into three groups: (i) dementia, cognitive decline, and behavior disturbances; (ii) movement and motor disturbances; (iii) combinations of both. The formation and deposition of physiochemically altered proteins, also known as misfolded proteins, into aggregates within the human brain are a common thread of NDs [4]. Specifically, the altered conformational structure of a protein results in its altered function or potentially toxic intra-/extra-cellular accumulation. The main misfolded proteins involved in most NDs include the following: (i) Amyloid-beta (Aβ), a peptide produced through the proteolytic cleavage of the transmembrane protein, amyloid precursor protein (APP). It accumulates in Aβ plaques, which are involved in the Alzheimer’s disease (AD) pathogenesis. (ii) α-synuclein (α-syn), a 140-aminoacid protein belonging to the family of synuclein proteins, highly expressed in presynaptic nerve terminals. It can accumulate in Lewy bodies, which are involved in synucleinopathies’ pathogenesis, such as Parkinson’s disease (PD). (iii) Microtubule-associated protein tau (MAPT). It promotes microtubule assembly and stabilization. Tau alterations due to genetic mutations or abnormal post-translational modifications, such as hyperphosphorylation, have been detected in NDs, known as tauopathies [5]. (iv) Prion protein (PrP), a glycosylphosphatidylinositol-anchored protein highly expressed on the cell surface of neurons [6]. It regulates peripheral nerve myelination homeostasis. The pathological form of PrP can accumulate into insoluble aggregates, causing the development of transmissible spongiform encephalopathies (TSEs), also known as prion diseases, including Creutzfeldt–Jakob disease (CJD). (v) TAR DNA-binding protein 43 (TDP-43), is a highly conserved nuclear RNA/DNA-binding protein involved in RNA-processing regulation. Abnormal post-translational modifications can lead to TDP-43 cytoplasmic accumulation and aggregation. Pathological TDP-43 aggregates can be found in several NDs, including amyotrophic lateral sclerosis (ALS) [7].

Misfolded proteins may deposit extracellularly, such as Aβ or PrP, or intracellularly, such as tau, α-syn, and TDP-43. Alterations in the levels of misfolded proteins in biological fluids, especially in the cerebrospinal fluid (CSF), can reflect the pathological mechanisms underlying NDs. Thus, they represent biomarkers of NDs.

A biomarker has been defined as “a characteristic that is objectively measured and evaluated as an indicator of normal biological processes, pathogenic processes, or pharmacologic responses to a therapeutic intervention” [8]. In the field of NDs, a biomarker is helpful for supporting definite diagnosis, detecting presymptomatic individuals, monitoring disease progression, and optimizing treatment strategies, offering the opportunity to appropriately select candidates for clinical trials [9]. Since many NDs share clinical features, detecting the underlying brain pathology is challenging. Biomarkers provide vital information on the underpinning mechanisms allowing for differential diagnosis. This is a critical issue for clinical trials. Indeed, misdiagnosis is a significant contributor to clinical trial failure.

Noteworthily, in the ND field, most biomarkers are measured in the CSF, which represents the ideal biological matrix for assessing neuropathological alterations since it communicates directly with the neuronal interstitium. The measurement of blood biomarkers is hampered by several issues, including the presence in the blood of proteolytic enzymes, antibodies, and other proteins, which could interfere with the detection, causing false results. Additionally, increased values could result from the expression of the biomarker by other organs and tissue. Finally, the biomarker concentration in blood may be too low to be detectable using the available analytical methods.

**Figure 1:**
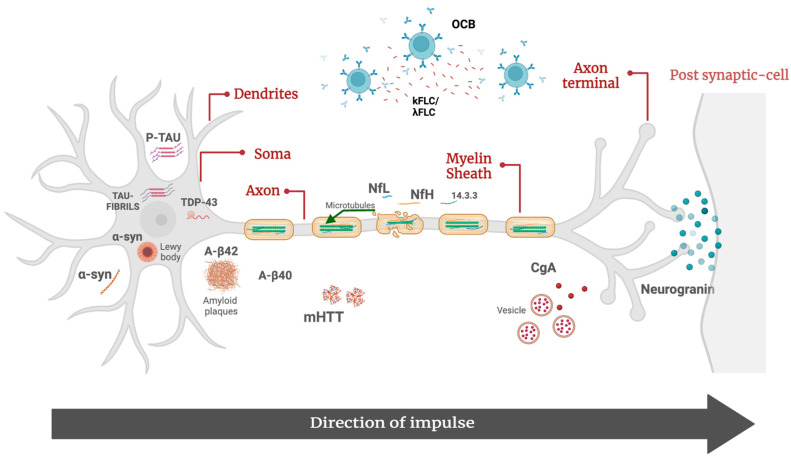
Molecular biomarkers of neurodegenerative disorders. Aβ42, β-amyloid 42; Aβ40, β-amyloid 40; p-tau, tau phosphorylated at threonine 181; α-syn, α-synuclein; CgA, chromogranin A; λFLC, free light-chain lambda; κFLC, free light-chain kappa; mHTT, mutated huntingtin protein; NfL, neurofilament lights; pNfH, phosphorylated neurofilament heavy; OCB, oligoclonal bands; TDP-43, TAR DNA-binding 43.

This study provides an overview on the molecular biomarkers of NDs currently used in clinical practice (Figure 1).

## 2. Molecular Biomarkers of Neurodegenerative Disorders

### 2.1. Alzheimer’s Disease

AD is the most common type of dementia worldwide, accounting for 60–80% of all cases [10]. Nowadays, dementia is a global health challenge, being the fifth-leading cause of death and AD the fourth-leading cause of disability-adjusted life-years (DALYs) lost in individuals aged 75 years and older [11]. Considering the aging population and the increased life expectancy, the burden of AD is expected to increase in the following years [12].

Clinically, it is characterized by altered cognition (memory loss), function (difficulty completing familiar tasks), and behavior.

Neuropathologically, it has two major features: (i) the extracellular deposition of Aβ42 peptide forming amyloid plaques in the neocortex of the brain; (ii) the intracellular deposition of phosphorylated-tau (p-tau) forming neurofibrillary tangles [13]. Other features include microglia activation, impaired synaptic function, blood–brain barrier (BBB) integrity, and reduced lipid transport and glucose metabolism.

Over the years, several hypotheses on AD pathogenesis have been proposed. Among these, the most reliable is the amyloid cascade formulated in the 1990s [14]. It postulates that an altered Aβ42 metabolism due to its increased production or impaired degradation results in the accumulation, oligomerization, and gradual deposition of Aβ42 oligomers as diffuse extracellular plaques. Plaques alter synaptic functionality and induce microglia and astrocyte activation, leading to an inflammatory response, which, in turn, promotes oxidative damage and the consequent altered activity of several enzymes, including kinases, which phosphorylate tau. The excessive phosphorylation of tau leads to neuronal tangle formation. All these alterations induce neuronal death and, consequently, neurodegeneration, leading to clinical symptom occurrence [15]. Thus, the amyloid cascade hypothesis relies on the idea that Aβ misfolding and deposition are the primary precipitants, which begin several decades before clinical manifestations. The progression from the initial neuropathological alterations to clinical symptoms is defined as the AD continuum [16]. On this continuum, there are three general phases: preclinical AD, mild cognitive impairment (MCI) due to AD, and dementia due to AD, also called Alzheimer’s dementia. The latter can be further divided into mild, moderate, and severe dementia. The AD continuum begins with preclinical AD, characterized by possible biological changes in the brain without symptoms, and ends with severe AD, characterized by severe symptoms [17]. The duration of each phase of the continuum varies among individuals. Specifically, the length of each part of the continuum is influenced by age, genetics, biological factors, sex, and other factors.

The diagnosis of AD is complex, being multistep and multidisciplinary, with the involvement of several professionals [18,19,20]. Laboratory medicine has a critical role in excluding secondary causes of dementia, such as thyroid dysfunction, anemia, and hyperglycemia, and in confirming the AD suspicion.

Biomarkers for excluding secondary causes of dementia include first-level lab tests, such as complete blood count, thyroid-stimulating hormone, glucose, vitamin D, vitamin B12, and electrolytes. Biomarkers for confirming AD suspicion include CSF Aβ42, Aβ 42/40 ratio, p-tau, and total-tau (t-tau) (Table 1). They represent the *core* biomarkers of AD, whose alterations reflect the pathological mechanisms underpinning the disease. The decrease in Aβ42 and Aβ 42/40 ratio levels indicates the presence of amyloid plaques, while an increase in tau proteins (t-tau and p-tau form) is associated with axonal loss and tau pathology.

While Aβ42 is sequestered within plaques, Aβ40 is not involved in the AD pathogenesis. However, since physiological interindividual variability in Aβ peptide levels exists, the ratio normalizes the inter-individual amyloid variations in the baseline CSF levels [21]. Overall, academic evidence suggests that evaluating the Aβ 42/40 ratio is superior to Aβ42 alone when identifying patients with AD [22]. Additionally, the Aβ 42/40 ratio has high concordance (>90%) with Aβ42-PET [23]. Finally, experts recommend CSF ratios to predict progression from MCI to AD [24].

The most recent criteria from the National Institute on Aging–Alzheimer’s Association (NIA-AA) and the International Working Group (IWG) recommend the measurement of CSF *core* biomarkers in the diagnostic work-up of AD [25,26,27]. Aβ42, Aβ 42/40 ratio, and p-tau alterations are specific to AD, while a t-tau increase can also be detected in other clinical conditions characterized by neurodegeneration. Typically, AD patients have decreased Aβ42 and Aβ 42/40 ratios and increased p-tau and t-tau levels. The only decrease in the Aβ42 and Aβ 42/40 ratio with normal p-tau and t-tau may be indicative of AD at early stages. Atypical biochemical profiles consistent with AD include altered Aβ42, Aβ 42/40 ratio, and t-tau, as well as altered Aβ42, Aβ 42/40 ratio, and p-tau [28]. In other words, the detection of an altered Aβ42 and Aβ 42/40 ratio, with or without altered tau, is indicative of the AD continuum.

To promote the clinical implementation of core AD biomarkers, fully automated assays have been developed. However, the CSF collection and handling hamper their widespread use. Indeed, CSF is collected by lumbar puncture, which is invasive and requires specialized personnel. Additionally, pre-analytical issues may be associated with false abnormal values [9]. On the other hand, CSF biomarkers have several advantages. First, they can detect brain changes at a very early stage. Further, they are less expensive than PET imaging (10–15-times lower) [22].

Recently, some methods to measure core biomarkers in blood have been developed. Indeed, recent advancements in both mass spectrometry and immunodetection methods have led to improved sensitivity, allowing for the detection of core biomarkers in plasma and serum. Ultrasensitive single-molecule array (Simoa) technology showed good accuracy for detecting Aβ40, Aβ42, t-tau, and p-tau in blood [29]. However, it has some limitations, hampering its widespread use in clinical practice, including high costs and dedicated and complex instrumentation that must be used by specialized personnel. Interestingly, in March 2023, Fujirebio launched immunoassays to measure blood core AD biomarkers using a fully automated platform. However, evidence of their reliability in clinical practice is still lacking [30].

However, they show less accuracy than their CSF counterparts. Blood-based biomarkers are very attractive in primary care, but further studies are required before implementing them in clinical practice.

Beyond core biomarkers, there is intensive research to identify reliable indicators of other pathological mechanisms involved in AD. In AD, synapse loss is the strongest pathological correlate of cognitive decline [31]. In the last few years, neurogranin (Ng), a postsynaptic protein, has gained much attention. It is a 78-amino-acid polypeptide, highly expressed in the hippocampus, with a critical role in long-term potentiation. Several authors showed that Ng levels are significantly increased in AD compared to healthy controls and patients with other NDs [32,33,34,35]. Ng may represent a biomarker of synaptic degeneration, which represents a distinct pathological event from amyloid deposition and neurofibrillary tangle formation. However, there is no consensus on its use in clinical practice. Additionally, no automated assays are available to measure its levels.

In the panorama of AD biomarkers, molecules indicative of neurodegeneration provide prognostic information. Among these, the most promising are neurofilament light chains (NfLs). NfLs are the most abundant and soluble subunit of neurofilaments, cylindrical proteins located exclusively in the cytoplasm of neurons, especially in the soma, dendrites, and axons. Neurofilaments are a key component of the cytoskeleton and play a critical role in maintaining the stability of axons and promoting conduction velocity.

Following axonal damage, neurofilaments are released into the CSF [36]. Thus, the NfL increase reflects axonal degeneration and injury. They are not specific to AD because their levels increase in several NDs, including multiple sclerosis (MS), ALS, and frontotemporal dementia (FTD) [37]. However, they provide useful information on neurodegeneration. Interestingly, their levels can be measured both in CSF and serum by fully automated assays. NfL levels are at a ratio of 1:40 blood/CSF [38]. Thus, NfLs are widely used in clinical practice.

### 2.2. Frontotemporal Dementia

FTD refers to a group of clinically, genetically, and pathologically heterogeneous NDs that affect the cortex of the frontal and temporal lobes, including paralimbic areas. It is regarded as a major cause of early-onset dementia worldwide [39] and, independently of age, is the third-most-common dementia after AD and Lewy body dementia.

Clinically, it is characterized by a spectrum of progressive alterations in social, behavioral, language, psychiatric, and motor aspects [40]. According to the last criterion, FTD is classified into behavioral-variant FTD (with a prevalence of behavioral alterations) and primary progressive aphasia, including the semantic variant, the nonfluent variant, and the logopenic variant [41].

The disease onset is generally around 50 years. However, FTD is characterized by a high rate of misdiagnosis, especially at a young age, because the symptoms can overlap with psychiatric disorders, such as schizophrenia. Further, 30–50% are familial, with several gene mutations identified. The pathological mechanisms underlying FTD are highly heterogeneous, with TDP-43 proteinopathies, followed by tauopathies, being the most common causes of familial FTD [42].

Despite significant strides in understanding the pathological mechanisms underpinning FTD, its diagnosis is still challenging. Nowadays, the FTD diagnosis relies on clinical and neuroradiological criteria [41]. AD core biomarkers are part of the diagnostic work-up to exclude AD. The definite diagnosis is made by detecting pathogenetic mutations or histopathological evidence of frontotemporal lobar degeneration. Nowadays, no specific biomarker for FTD is available. Current research shows interesting findings for some biomarkers. NfLs emerged as a reliable indicator of disease severity [43]. Additionally, some authors showed that NfLs are significantly increased in FTD patients than in other NDs, such as AD and PD, opening the possibility to use them for differential diagnosis [39]. However, further studies to establish the decisional cut-off are mandatory. Another interesting aspect is that NfLs may be helpful for differentiating FTD and psychiatric disorders, which usually do not show an increase in NfLs levels.

A combined use of NfLs and the Aβ42 and Aβ 42/40 ratios has been proposed for distinguishing AD and FTD; the increase in NfLs associated with normal Aβ42 and Aβ 42/40 ratio may be indicative of FTD.

### 2.3. Parkinson’s Disease

PD is the second-most-common ND, affecting 2–3% of the population ≥65 years of age, and one of the leading causes of neurological disability worldwide [44]. Clinically, it is characterized by progressive motor symptoms over time, such as bradykinesia, rigidity, rest tremor, postural instability, and gait disturbances, including dysphagia. Additionally, non-motor symptoms, including hyposmia, cognitive decline, sleep disturbances, urinary dysfunction, and constipation, are common. PD is characterized by high heterogeneity in terms of age of onset, clinical presentation, and progression rate.

The neuropathological hallmarks of PD are intracellular inclusions consisting of aggregated and misfolded α-syn, known as Lewy bodies and Lewy neurites, and striatal dopamine deficiency due to neuronal loss in the substantia nigra [45].

The underlying molecular pathogenesis involves multiple pathways and mechanisms, such as α-syn proteostasis, mitochondrial dysfunction, oxidative stress, altered calcium homeostasis, axonal transport, and neuroinflammation.

PD diagnosis is challenging, especially in the early stage, because it presents signs and symptoms similar to atypical Parkinsonism disorders, including multiple system atrophy, progressive supranuclear palsy, and corticobasal degeneration [46]. Other common misdiagnoses include non-Parkinson’s disease, tremor disorders, and secondary Parkinsonism. An error rate in PD diagnosis up to 35% has been reported [47].

Nowadays, the diagnosis is exclusively based on clinical criteria, and no biomarker has been recommended by guidelines [48]. A promising candidate is α-syn, which has a primary role in PD pathogenesis. Noteworthily, it is expressed by different tissues, and it has a bidirectional movement between blood and brain [49]. Thus, an alteration in its levels may not be the result of PD pathology. However, several studies indicate that the overall levels of α-syn in the CSF are lower in PD patients compared to healthy controls [50,51,52,53,54]. A 2017 meta-analysis reported that the sensitivity and specificity for distinguishing PD from controls were 0.72 and 0.65, respectively, for total α-syn [55].

α-syn has also been investigated in other biological matrices, such as serum and plasma. A 2022 meta-analysis suggests that the increase in plasma/serum levels of total α-syn in PD is primarily observed in the early stages of the disease. This increase appears to be significantly associated with younger age, shorter disease duration, mild motor impairment, and an immunomagnetic reduction test for protein quantification [56]. Thus, these findings suggest a potential prognostic use of plasma/serum α-syn.

NfLs are also potential prognostic biomarkers of PD [57,58]. Several authors described very consistent associations between serum NfL and motor progression and cognitive worsening [59]. Thus, serum NfLs could serve as an easily accessible biomarker to assess the severity and progression of motor decline in PD patients [60].

### 2.4. Dementia with Lewy Bodies

Dementia with Lewy bodies (DLB) is a chronic and degenerative neurological condition characterized by the accumulation of the α-syn in Lewy bodies located in the cytoplasm of cortical neurons. It is the third-most-common form of dementia worldwide, following AD and vascular dementia. DLB shares features with both AD and PD, involving cognitive and motor alterations [61].

Diagnosing DLB can be complex because its symptoms overlap with other clinical conditions. The McKeith diagnostic criteria for DLB provide helpful guidelines to identify it [62]. However, no tests can definitively diagnose DLB [63]. Even though not yet established as a biomarker, α-syn can be helpful in distinguishing between DLB and AD, with increased levels indicating AD [64]. Additionally, Aβ, t-tau, and p-tau could be helpful in determining concomitant AD pathology or predicting cognitive decline.

### 2.5. Creutzfeldt–Jakob Disease

CJD is a rapidly progressive fatal rare ND, classified as transmissible spongiform encephalopathy (TSE) or “prion disease”, affecting both humans and other mammalian species [65]. Among humans, CJD is the most common prion disease. CJD was first described in 1920 by Hans Creutzfeldt and later in 1921 and 1923 by Alfons Jakob. According to the mode of transmission, CJD is classified into three forms: sporadic (sCJD), which is the most common, accounting for 85–90% of cases; genetic, accounting for 15% of cases, due to autosomal-dominant mutations in the prion protein gene (PRNP); and acquired, accounting for less than 1% of cases resulting from prion transmission by an external source, such as the human exposure to bovine spongiform encephalopathy (BSE) during the late 1980s and early 1990s [66].

Globally, the incidence of CJD is typically around 1–2 cases per million per year [67]. Clinically, it presents with rapidly progressive dementia and early myoclonus and ataxia. Noteworthily, it has a long asymptomatic incubation period, ranging from 1 to 42 years, and most patients die within a year of clinical onset.

It is caused by the transformation of a normal cellular prion protein (PrP^c^) into a misfolded, transmissible proteinaceous infectious particle, also known as PrP scrapie (PrP^Sc^). PrP is a normal neuron protein, with a predominantly α-helical and random coil composition. PrP^Sc^ are self-propagating proteins reproducing by interacting with normal PrP cellular isoforms, converting α-helices into indigestible β-pleated sheets. The highly chemically stable β-pleated aggregates cause derangements in intracellular protein folding, ubiquitination, and trafficking in affected neurons, leading to neurodegeneration [68].

Prompt and accurate diagnosis of CJD is fundamental for intervention strategies and overall outcomes. The diagnosis is challenging because it must be differentiated from other clinical conditions characterized by rapidly progressive dementia (RPD). The integration of clinical, laboratory, imaging, and electroencephalogram (EEG) findings guide the CJD diagnosis. Among laboratory tests, the detection of 14-3-3 protein and very high levels of t-tau (greater than 1150 pg/mL) supports the CJD diagnosis (Table 1), although they are not specific to the disease [69].

Of note, case–control studies showed that 14-3-3 protein allows for differential diagnosis between CJD and other NDs, such as AD, DLB, and FTD. However, the specificity of CSF 14-3-3 is reduced when control groups include events of acute neuronal damage and inflammatory and infiltrative neoplastic diseases of the CNS [70,71]. Conversely, most studies reported a good sensitivity and specificity of CSF t-tau, each around 90% [69,72]. Some authors agree that t-tau is a better diagnostic marker than 14-3-3, leading to ongoing discussion and controversy over which biomarker should be primarily used. Unfortunately, there is no consensus on the best t-tau cut-off for CJD diagnosis [73,74]. The p-tau/t-tau ratio is an important alternative biomarker for CJD, with very high diagnostic accuracy in differentiating CJD from other neurological diseases.

Excellent diagnostic accuracy has also been reported for NfLs in distinguishing healthy individuals from CJD patients. However, NfLs have low specificity, being increased in several NDs [75,76]. Similarly, α-syn increases in CJD patients, likely due to rapid neurodegeneration. A multicenter study demonstrated excellent diagnostic accuracy in discriminating CJD from other NDs (including dementia syndromes) at an optimal cut-off of 820 pg/mL [77].

Finally, genetic analysis of PRNP should be considered in all suspected cases of CJD to determine codon 129 polymorphism and exclude pathogenic mutations, which may be present in patients with a negative family history. It is important to emphasize that routine blood, CSF, and imaging diagnostics should always be performed to exclude more common differential diagnoses [78].

### 2.6. Amyotrophic Lateral Sclerosis

ALS is a devastating disease, first described by Charcot in 1874 [79]. ALS affects approximately 2 out of 100,000 people and is characterized by the progressive loss of upper motor neurons in the cortex and lower motor neurons in the brainstem and spinal cord. Symptoms include muscle atrophy, speech, swallowing difficulties, fasciculations, and spasticity. The prognosis is severe, with a median survival of 2–5 years from diagnosis, often due to respiratory complications [80]. ALS is considered a proteinopathy due to protein aggregates in the affected neurons. While most cases are sporadic (sALS), a small percentage are familial (fALS), with both forms sharing similar pathological features, such as protein inclusions and neurological symptoms. However, there are differences among patients with different mutations. More than 50 genes associated with ALS have been identified, with mutations in the SOD1, TARDBP, FUS/TLS, and C9ORF72 genes being more common in familial forms [81]. These genes are involved in various cellular processes, including mitochondrial dysfunction, excitotoxicity, autophagy with protein homeostasis loss, inflammation, DNA damage repair, aberrant RNA metabolism, and compromised intracellular trafficking [82]. ALS can be distinguished based on the involvement of upper or lower motor neurons, resulting in different clinical onsets, i.e., bulbar or spinal [80]. According to the latest Gold Coast criteria, the diagnosis is fundamentally clinical. Molecular biomarkers can aid in diagnosis and differentiate ALS from similar conditions [83]. The latest diagnostic criteria from the Gold Coast rely on the evaluation of nerve conduction and electromyography (EMG); magnetic resonance imaging (MRI); and measurement of blood and/or CSF biomarkers [84,85]. Although CSF is a primary source of biomarkers, it can be challenging to obtain in advanced ALS patients. Therefore, biomarkers in blood or urine may be more practical in the later stages of the disease. While there are no guidelines yet for circulating biomarkers in early ALS diagnosis, research on potential biomarkers, such as NfL, microRNA (miRNA), and TDP-43, in blood shows promise as prognostic indicators. Most studies have examined the levels of phosphorylated neurofilament heavy (pNfH) and NfL in CSF, finding them significantly elevated in ALS, with sensitivity and specificity exceeding 80% [86,87]. Interestingly, serum NfL can differentiate ALS from other NDs. Currently, only patients with Guillain-Barré syndrome (GBS) and CJB disease have shown serum NfL levels comparable to those observed in ALS. However, these conditions are clinically distinct from ALS, making misdiagnosis unlikely. The ratio of CSF p-tau/t-tau may identify ALS patients, with an area under the curve of 0.78 [88]. Additionally, numerous studies are considering the use of non-coding miRNAs as biomarkers for ALS diagnosis. A recent study on blood miRNAs identified miR-181 as a robust prognostic marker in ALS [89]. Patients with elevated plasma levels of miR-181 had nearly five-times higher odds of dying during the study period. Combining blood miR-181 measurement with NfL further strengthened prognostic performance in ALS. TDP-43 in blood has also shown promise as a prognostic biomarker. In a study on sALS patients, the plasma levels of TDP-43 were positively correlated with patients’ generalization time, defined as the interval between reported disease onset and the appearance of bulbar and spinal involvement signs [90]. These data suggest that higher levels of TDP-43 in plasma may be observed in a slower disease progression, likely due to increased hematological clearance of this pathological protein, reflecting a reduced pathological burden of TDP-43 in the brain. Research on circulating biomarkers, such as NfL, miRNA, and TDP-43, in blood shows significant promise in aiding the early diagnosis and prognosis of ALS, offering potential tools to differentiate ALS from other neurodegenerative conditions and better understand disease progression.

### 2.7. Multiple Sclerosis

MS is a chronic inflammatory autoimmune demyelinating disease of the central nervous system (CNS). It is characterized by various neurological symptoms, including vision problems, coordination difficulties, cognitive alterations, and muscle weakness. It typically presents in young adults but can occur at any age, including childhood [91]. There are approximately 2.8 million individuals worldwide living with MS, and the incidence and prevalence rates are higher in women, with a women/men ratio varying 3:1 [92].

The disease can be classified into three main types: relapsing–remitting MS (RRMS), primary progressive MS (PPMS), and secondary progressive MS (SPMS). Additionally, the concept of clinically isolated syndrome (CIS) includes patients experiencing their first clinical episode but not yet meeting all diagnostic criteria for definitive MS. However, CIS patients are highly likely to progress to the definite form of the disease over time [93].

Currently, the diagnosis of MS is made according to the McDonald criteria revised by AJ Thompson in 2017, including a clinical neurological examination, the presence of oligoclonal bands (OCBs) in the CSF indicative of intrathecal IgG synthesis, MRI findings, and differential diagnosis to exclude other diseases [94,95]. OCBs are detected through isoelectrofocusing (IEF), which separates molecules according to their charge, molecular weight, and isoelectric point. This technique has been approved by the Food and Drug Administration (FDA) for MS diagnosis [96].

The basic concept for diagnosing MS involves the presence of Dissemination in Space (DIS), which means the development of lesions in distinct anatomical areas within the CNS. Additionally, diagnosis requires monitoring the Dissemination in Time (DIT), which involves the development of new lesions over time.

Beyond OCB, other biomarkers for supporting the MS diagnosis include free kappa (κFLC) and lambda (λFLC) light chains, cytokines, and NfL [97].

The quantification of CSF κFLCs and λFLCs levels provides prognostic information (Table 1). κFLCs and λFLCs, produced during antibody synthesis by B cells, can be detected in both blood and CSF and have shown higher sensitivity than IgG for MS diagnosis [98]. Elevated CSF κFLC levels are associated with conversion from CIS to MS and disability extent in MS patients. On the other hand, λFLCs have shown good sensitivity in detecting the intrathecal production of immunoglobulins in individuals with CNS inflammatory disorders. However, the current McDonald criteria do not yet include quantifying κFLCs as a potential diagnostic biomarker [99,100,101].

Lastly, serum NfL levels are increased in all stages of MS and can be considered a useful biomarker for monitoring disease activity, predicting progression, and assessing treatment responses [102,103,104]. Also, glial fibrillary acidic protein (GFAP), an intermediate astrocyte cytoskeletal protein indicating astrocyting activation, has been recently shown to be higher in progressive MS than in RRMS and correlate with disability. However, further studies are required to define their clinical usefulness.

Recent research has focused on miRNA as potential biomarkers for MS. These small RNA molecules can be detected in various bodily fluids and seem to play a role in the development of MS. For example, miR-20a-5p has been found to be under-regulated in patients with RRMS, PPMS, and SPMS, while miR-22-5p is over-regulated in the CSF and blood of MS patients. However, confirmation on larger cohorts is needed to validate these findings [105,106].

### 2.8. Huntington’s Disease

Huntington’s disease (HD) is a rare ND of the CNS, characterized by involuntary chorea movements, behavioral disturbances, psychiatric alterations, and dementia.

The incidence of HD varies among populations, but it is considered rare, affecting around 5–10 people per 100,000 worldwide. The disease onset is generally between 30 and 50 years [107].

The underlying pathobiological mechanisms of HD are complex and involve various mechanisms. At the core of the pathology is an autosomal-dominant mutation in the huntingtin gene (HTT) located on chromosome 4, resulting in a toxic form of the huntingtin protein (mHTT). The accumulation of this protein leads to progressive degeneration of brain neurons [108].

The presence of mHTT is a hallmark of the disease. Thus, research focused on quantifying mHTT as a biomarker for diagnosing the disease and evaluating the effectiveness of pharmacological treatments. Several methods have been developed to detect mHTT in biological fluids, such as blood and CSF, including sensitive techniques like Förster resonance energy transfer (TR-FRET) and single-molecule counting immunoassays (SMCs). Studies have shown that elevated levels of mHTT in CSF are associated with disease severity and the likelihood of onset in premanifest individuals [109].

In addition to mHTT, several potential biomarkers have been evaluated, such as S100 calcium-binding protein B (S100B), NfL, and tau protein. However, many of these molecules have not shown significant differences between HD patients and healthy controls and did not correlate with disease severity [110,111].

NfL has emerged as a promising HD biomarker, with elevated CSF levels associated with disease severity and progression. Furthermore, NfL levels in plasma have been associated with disease progression and can predict the onset of the disease in premanifest mutation carriers [112].

Tau protein also appears to be involved in the pathology of HD. Abnormal forms of tau have been found in various brain structures of HD patients, like those seen in AD. Studies have highlighted that tau accumulation in the brains of HD patients can be toxic and correlate with disease progression. In CSF studies, elevated tau concentrations have been observed in HD patients, with correlations between tau levels and cognitive and motor deficits, indicating a potential role for tau in CSF as a biomarker for HD-related psychiatric symptoms [113].

Given the involvement of mHTT in leukocytes and microglia, several studies have focused on immune biomarkers to monitor HD patients and evaluate the effectiveness of immunomodulatory treatments. Various immune biomarkers, such as IL-6 and CSF YKL-40, have been identified, showing correlations with the disease stage and severity of motor and functional symptoms. However, questions remain about these immune system alterations’ primary or secondary nature and their utility in clinical trials [114,115].

So far, none of the discussed biofluid biomarkers have been clinically validated. However, the closest to validation in HD are assays for HTT and protein markers of neuronal damage like NfL, which are already useful as exploratory endpoints.

### 2.9. Spinal Muscular Atrophy

Spinal muscular atrophy (SMA) is a genetic disease affecting motor neurons in the spinal cord, leading to progressive muscle degeneration and subsequent muscle weakness [11]. It affects approximately 1 in 10,000–20,000 live births. Despite its low incidence, SMA has the highest rate of infant mortality among genetic neuromuscular diseases [116]. The disease is primarily caused (95% of cases) by a homozygous deletion in the SMN1 gene, which encodes for the Survival Motor Neuron protein (SMN), essential for motor neuron maintenance. The remaining cases mainly result from a heterozygous deletion in one allele and a point mutation in the second allele. Patients with SMA have a variable number of copies of a second gene, called SMN2, which produces a shortened form of the SMN protein with reduced functionality compared to the full-length SMN protein produced by the wildtype SMN1 gene. Consequently, the varying number of SMN2 gene copies is responsible for the disease’s wide range of clinical manifestations, which can vary in severity and symptoms [116,117].

According to the Consensus Statement for Standard of Care in Spinal Muscular Atrophy, the first diagnostic test for patients with suspected SMA should be genetic analysis for deletions in the SMN gene. If such analysis is negative, further laboratory tests should be performed, including muscle enzyme creatine kinase (CK) and electrophysiological tests, such as EMG and repetitive nerve stimulation studies. If EMG suggests a motor neuron disease, additional tests for SMN mutations should be pursued. If the patient has only one copy of SMN1, the remaining copy may contain subtle mutations, such as point mutations, insertions, or deletions. Sequencing of the coding region of the remaining SMN1 copy can identify the mutations and confirm the diagnosis of SMA 5q. If the patient has two copies of SMN1, consideration should be given to other motor neuron disorders [118].

Recently, NfL has emerged as a promising biomarker of neuroaxonal damage in SMA, with elevated levels reflecting the severity of the condition. Moreover, it has been observed that NfL levels decrease rapidly after implementing effective therapies [119,120,121,122].

Other biomarkers may have a prognostic role in SMA, such as the number of SMN2 copies that significantly influences disease severity, being associated with less SMN protein and a more severe/advanced disease [123]. Early proposed circulating prognostic biomarkers in SMA were serum creatinine and CK. In rapidly progressive forms of SMA, serum creatinine levels are inversely associated with disease severity [119]. It is also higher in patients with chronic forms than those with the rapidly progressive form [124]. An increase in CK levels has been described, especially in chronic forms. In pediatric and adult patients treated with Nusinersen, a decrease in CK and a parallel increase in serum creatinine have been observed after several months of treatment [125]. Therefore, these two biomarkers could be helpful for monitoring treatment progression and response.

### 2.10. Spinocerebellar Ataxia

Spinocerebellar ataxia (SCA) is a group of NDs with autosomal-dominant transmission, primarily involving the cerebellum. Symptoms include alterations in voluntary movement coordination, difficulty walking, balance issues, and, sometimes, language and vision disturbances. Onset typically occurs in adulthood. There are several variants of SCA, each caused by specific genetic mutations. These variants are designated with the prefix SCA followed by a number, indicating the order of identification of the genetic mutations. Some of the most common types include the following: spinocerebellar ataxia type 1 (SCA1), spinocerebellar ataxia type 2 (SCA2), spinocerebellar ataxia type 3, also known as Machado-Joseph disease (SCA3/MJD), spinocerebellar ataxia type 6 (SCA6), spinocerebellar ataxia type 7 (SCA7), spinocerebellar ataxia type 8 (SCA8), spinocerebellar ataxia type 10 (SCA10), and spinocerebellar ataxia type 12 (SCA12). SCA3 is the most common autosomal-dominant cerebellar ataxia worldwide, and it is caused by a mutation in the ATXN3 gene [126].

The genetic basis of SCAs is heterogeneous; the most common SCAs are caused by the expansion of CAG trinucleotide repeats, which encode a polyglutamine tract in the protein product (e.g., SCA3/MJD). Polyglutamine SCAs account for over half of the known SCAs and are the best characterized. Others are caused by nucleotide repeats, but these repeats do not occur in the coding region of the gene (e.g., SCA8). In these cases, it is believed that the pathology arises from toxic RNA species or a peptide product resulting from a non-canonical form of translation, known as repeat-associated non-ATG translation (RAN translation). The remaining SCAs are caused by conventional mutations, including point mutations or deletions in the coding regions of genes, producing abnormal proteins [127].

The guidelines published in the Orphanet Journal of Rare Diseases in 2019 provide a detailed overview of recommendations for diagnosing and managing progressive ataxias, such as SCA. They emphasize the importance of thorough medical history-taking, comprehensive clinical examination, and the use of relevant investigations for effective management, considering the various manifestations of ataxias. Key elements in medical history include the onset speed of symptoms, age of onset, and family history. Patients often report incoordination, instability, clumsiness, and language confusion. Diagnostic investigations range from blood tests to advanced genetic testing, such as next-generation sequencing, and also include neuroimaging and nerve conduction studies. Generic and specific assessment scales are available to monitor disease progression and evaluate therapeutic interventions, essential for both clinical research and practice [128].

In the context of SCAs, the definitive diagnosis relies on genetic testing.

Biomarkers, sought after to predict the transition from asymptomatic to symptomatic phases or to provide insights into the progression of the disease, are strongly pursued. Although no validated biomarker has been identified for SCAs yet, there are some promising candidates, such as neurofilaments, which provide prognostic information, and ataxin [129,130,131,132]. However, more studies are mandatory to establish their usefulness in clinical practice.

## 3. Practical Recommendation for Blood and CSF Collection and Processing for ND Biomarker Analysis

This section provides helpful information regarding the management and analysis of ND biomarkers, divided into three phases: pre-analytical, analytical, and post-analytical.

### 3.1. Pre-Analytical Phase

To appropriately use ND biomarkers, it is crucial to understand the importance of pre-analytical variables.

**Figure 2:**
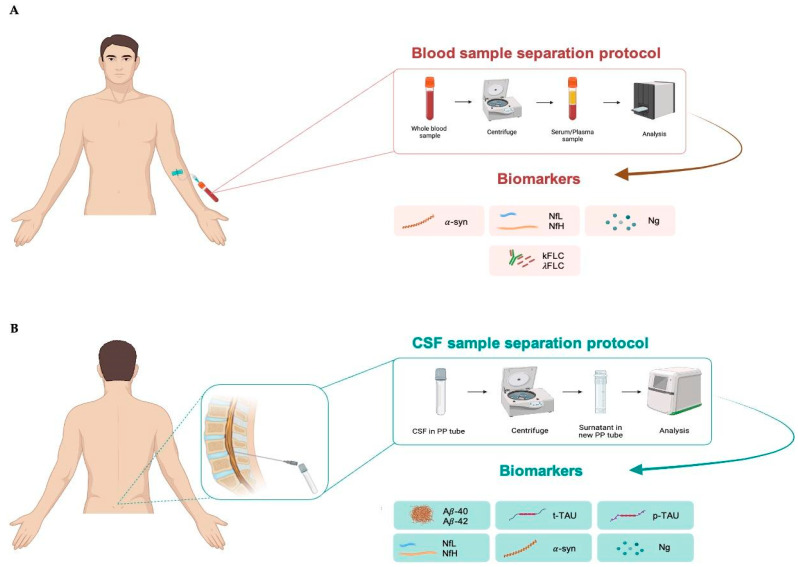
Blood (**A**) and cerebrospinal fluid; (**B**) sample separation protocol. CSF, cerebrospinal fluid; α-syn, α-synuclein; NfL, neurofilament lights; NfH, neurofilament heavy; Ng, neurogranin; κFLC, free light-chain kappa; λFLC, free light-chain lambda; Aβ40, β-amyloid 40; Aβ42, β-amyloid 42; t-tau, total tau; p-tau, tau phosphorylated at threonine 181.

For blood sample collection, it is recommended to use EDTA tubes to separate plasma or non-anticoagulated tubes to separate serum. After collection, the sample is centrifuged at 2500 rpm for 10 min to separate plasma/serum from the cellular component. Subsequently, the plasma/serum is transferred to collection tubes, ready for analysis (Figure 2A), and can be stored at −30 °C for short or at −80 °C for longer periods.

CSF is collected via lumbar puncture in the L3–L4 region using a sterile polypropilene syringe to avoid the risk of infection. It is recommended to use polypropylene (PP) tubes for collecting CSF. Further, 94% of laboratories use PP tubes to maintain sample integrity and prevent contamination or interactions with the tube material. The use of PP is especially recommended for Aβ analysis because this peptide is sticky. Finally, PP tubes are resistant to extreme temperatures, making them appropriate for storing CSF samples at −30 °C and −80 °C for long periods [133]. After collection, the CSF sample should be centrifuged at 1000 rpm for 5 min to separate cellular components, such as red blood cells and immune cells, from the liquid fraction. Blood contaminations from tissue trauma should be avoided, especially for Aβ analysis, because proteins such as albumin and proteases found in blood may interact with or break down Aβ, while blood cells themselves contain significant amounts of Aβ42. Thus, protocols for CSF collection consistently advise discarding the initial 1–2 mL of CSF in the presence of visible blood contamination or if the CSF sample contains more than 50 erythrocytes per μL. The sample ready for analysis can be stored at −30 °C for short or at −80 °C for longer periods (Figure 2B).

The evidence regarding the impact of freeze/thaw cycles on biomarker concentration is inconclusive. However, the current protocols recommend up to three cycles. Indeed, it has been shown that increasing the number of freeze/thaw cycles decreases CSF concentrations of biomarkers, but evidence regarding the exact number of cycles remains inconclusive.

Overall, it is crucial to pay attention to pre-analytical variables, as these can significantly impact the quality and reliability of ND biomarker analysis results.

### 3.2. Analytical Phase

When analyzing ND biomarkers, various factors can affect the accuracy and reproducibility of results. The techniques commonly used in most labs are chemiluminescence, ELISA, and Simoa.

Furthermore, 76% of laboratories employ Fujirebio immunoassays (Lumipulse) for quantifying Aβ40, Aβ42, t-tau, and p-tau levels. Conversely, other markers, such as NfL, α-syn, and Ng, are measured using chemiluminescence, ELISA, or the Simoa method [133,134].

Firstly, it is crucial to ensure standardized and high-quality reagents, stored properly and used according to the manufacturer’s instructions. Additionally, accurate instrument calibration is essential for obtaining precise measurements. Equally important is implementing quality controls to monitor the accuracy and precision of the test. Conducting repeatability and reproducibility tests is another necessary step to assess the consistency of results across different test runs and operators in the laboratory. Additionally, correct processing of samples throughout all stages of analysis is essential to avoid contamination and degradation.

Last, validating the analysis method is fundamental to confirm its precision, accuracy, and sensitivity in detecting the biomarkers of interest. This process should involve determining the detection limit, quantification limit, and linearity of the test.

### 3.3. Post-Analytical Phase

The post-analytical phase is also crucial for appropriately interpreting results.

Firstly, a comprehensive evaluation of the obtained results is essential, considering both the values of individual biomarkers and any relationships between them. A concrete example of this post-analytical phase is the use of Aβ40 to normalize the value of Aβ42, due to interindividual differences in the expression of this protein. This approach accounts for individual variations and provides a more accurate assessment of Aβ levels in the analyzed sample.

In the report, along with standard reference ranges, an interpretative note should be included to guide clinicians in correctly interpreting the lab result, especially for uncommon biomarkers. It provides information on the clinical importance of biomarkers and their relationship with the patient’s health status. This helps clinicians make informed diagnostic and therapeutic decisions, thereby improving patient care.

Subsequently, the clinician must integrate the lab results with the other patients’ findings to make diagnostic and therapeutic decisions.

## 4. Challenges and Perspectives

Reliable biomarkers that can precisely indicate disease activity, aid in diagnosis, and monitor the progression of neurodegenerative diseases are essential for the advancement of effective therapies. Currently, CSF represents the most reliable biological matrix to measure biomarkers of NDs. However, CSF has significant limitations, mainly due to the collection method. Thus, intensive research is ongoing to find alternative, reliable biofluids. In the last few decades, major advancements have been made in the development of blood-based biomarkers for several neurodegenerative diseases. However, studies to assess their accuracy and the potential factors influencing their levels are ongoing, and more efforts are required before introducing them to routine clinical laboratories.

Unlike other non-communicable diseases, most NDs lack a cure, and there are limited treatment options available. Present approaches encompass medications, physical and occupational therapy, speech therapy, assistive devices, and lifestyle modifications, all of which can assist in managing symptoms and decelerating the progression of the disease.

It is imperative to recognize that while these treatments enhance symptoms and improve quality of life, they do not halt or reverse the progression of the disease. The primary obstacle in identifying preventive and curative therapeutics lies in the existing gap in scientific understanding regarding the early molecular events that are crucial for disease initiation. Progress in proteomics, transcriptomics, and metabolomics has yielded valuable insights into the mechanisms underlying these disorders, paving the way for the exploration of innovative diagnostic and therapeutic strategies.

## 5. Conclusions

Nowadays, NDs represent a significant global public health issue, with an incidence progressively increasing over the years, partially due to an aging population. The diagnosis and the entire path of care of NDs are challenging because symptoms often overlap among different disorders.

Laboratory medicine provides precious tools to support clinicians in the appropriate detection and management of NDs. Biomarkers have revolutionized the AD field, and intensive efforts are ongoing to improve the whole path of care for other NDs, with the aim to ameliorate the patients’ quality of life and reduce the social and economic load associated with these conditions.

## Figures and Tables

**Table 1:** Diagnostic and prognostic biomarkers in neurodegenerative diseases.

Disease	Biological Matrix	Biomarkers	
		Diagnosis	Prognosis
Alzheimer’s disease continuum	CSF	↓ Aβ42 and Aβ2 42/40 ratio ↑ p-tau ↑ t-tau	↑ Ng ↑ NfL
		↓ Aβ42 and Aβ2 42/40 ratio N p-tau and t-tau	
		↓ Aβ42 and Aβ2 42/40 ratio ↑ p-tau N t-tau	
		↓ Aβ42 and Aβ2 42/40 ratio N p-tau ↑ t-tau	
	Blood	N.A.	↑ NfL
Frontotemporal Dementia	CSF and blood	N.A.	↑ NfL
Parkinson’s Disease	CSF	↓ α-syn ↑ NfL	N.A.
	Blood		↑ NfL
Dementia with Lewy bodies	CSF	↓ Aβ42 and Aβ2 42/40 ratio ↑ p-tau ↑ CgA	↑ α-syn ↑ Ng
Creutzfeldt-Jakob disease	CSF	↑ PrP ↑ 14-3-3 ↑ t-tau ↓ p-tau/t-tau	↑ NfL ↑ α-syn
	Blood	↑ NfL	N.A.
Amyotrophic Lateral Sclerosis	CSF	↑ TDP-43 ↑ NfL ↑ pNfH ↑ t-tau ↓ p-tau/t-tau	N.A.
	Blood	N.A.	↑ TDP-43 ↑ NfL
Multiple sclerosis	CSF	↑ OCB ↑ λFLC ↑ κFLC	↑ NfL
	Blood	↑ λFLC ↑ κFLC	↑ NfL
Huntington’s disease	CSF	↑ mHTT	↑ NfL
	Blood	N.A.	↑ NfL
Spinal muscular atrophy	CSF	N.A.	↑ NfL
	Blood	N.A.	↑ NfL ↑ pNfH
Spinocerebellar ataxia	CSF	N.A.	↑ p-tau ↑ t-tau
	Blood	N.A.	↑ NfL ↑ p-tau ↑ t-tau

CSF, cerebrospinal fluid; N, normal; Aβ42, β-amyloid 42; Aβ40, β-amyloid 40; t-tau, total tau; p-tau, tau phosphorylated at threonine 181; Ng, neurogranin; α-syn, α-synuclein; NfL, neurofilament lights; pNfH, phosphorylated neurofilament heavy; CgA, chromogranin A; PrP, prionic protein; TDP-43, TAR DNA-binding 43; OCB, oligoclonal bands; λFLC, free light-chain lambda; κFLC, free light-chain kappa; mHTT, huntingtin protein. N.A. not available.

## Data Availability

Not applicable.
